# Predictive Equations Overestimate Resting Metabolic Rate in Young Chilean Women with Excess Body Fat

**DOI:** 10.3390/metabo13020188

**Published:** 2023-01-27

**Authors:** Eduard Maury-Sintjago, Alejandra Rodríguez-Fernández, Marcela Ruíz-De la Fuente

**Affiliations:** 1 Department of Nutrition and Public Health, Universidad del Bío-Bío, Chillan 3780000, Chile; 2 Auxology, Bioanthropology, and Ontogeny Research Group (GABO), Faculty of Health and Food Sciences, Universidad del Bío-Bío, Chillan 3780000, Chile

**Keywords:** resting metabolic rate, predictive equations, indirect calorimetry, women, body fat

## Abstract

Underestimating/overestimating resting metabolic rate (RMR) affects energy prescription. The objective was to compare RMR by indirect calorimetry (RMR IC) and RMR estimated by predictive equations in women with excess body fat. This was an analytical cross-sectional study with 41 women aged 18–28 with overnutrition according to body composition. The RMR IC was measured and RMR estimated using the FAO/WHO/UNU (1985), FAO/WHO/UNU (2004), Harris–Benedict, and Mifflin–St Jeor equations. The percentage of adequacy (90–110%), overestimation (>110%), and underestimation (<90%) were evaluated for RMR IC. Data were described by percentiles because of non-normal distribution according to the Shapiro–Wilk test. The Kruskal–Wallis test and Bland–Altman analysis were applied at a significance level of α < 0.05. The RMR IC was 1192 and 1183 calories/day (*p* = 0.429) in women with obesity and overweight, respectively. The FAO/WHO/UNU (1985), FAO/WHO/UNU (2004), Harris–Benedict, and Mifflin–St Jeor equations overestimated the RMR IC by 283.2, 311.2, 292.7, and 203.0 calories/day and by 296.7, 413.8, 280.0, and 176.6 calories/day for women with overweight and obesity (*p* < 0.001), respectively. The Harris–Benedict adjusted weight (0.5) equation underestimated RMR IC by 254.7 calories/day. The predictive equations overestimated RMR IC in women with excess body fat. The Mifflin–St Jeor equation showed less overestimation and better adequacy, but was not exempt from inaccuracy.

## 1. Introduction

Total energy expenditure consists of three principal components, which are the basal metabolic rate (BMR), the thermic effect of food, and physical activity [1]. The BMR is the minimal energy required to maintain critical body functions, and it represents approximately 60% to 75% of the total energy expenditure in individuals with a sedentary lifestyle [2]. It is determined based on body size, age, sex, hormones [3], body fat [4], and genetic traits [5], and fat-free mass is its main determinant [6]. The concepts of BMR and resting metabolic rate (RMR) are often used interchangeably but there is a difference of at least 10% between RMR and BMR [7].

The RMR must be estimated in a nutritional dietary intervention to prescribe an ad-equate diet in terms of calories and nutrients. There are various methods to measure RMR, and indirect calorimetry (IC) is considered as the gold standard [8]. However, its application is limited because it is costly, time-consuming, and requires trained personnel to take the measurements [8]. The IC method calculates the individual’s RMR by measuring the oxygen (O_2_) and carbon dioxide (CO_2_) concentrations in the expired air [9].

In routine clinical practice, RMR is estimated using different predictive equations, which have proven to be easy to use, cost-free, and always available. They are derived from regression models that include sex, age, anthropometric measurements (weight and height), and body composition (lean body mass and body fat) variables. Among the most used equations are the Harris–Benedict [10], FAO/WHO/UNU [11], and Mifflin–St Jeor [12]; the latter is recommended by the American Dietetic Association (ADA) [13].

The accuracy of the predictive equations is determined by the individual’s nutritional status. Predictive formulae have been less accurate in adults with excess body fat [14]. Several studies have shown that estimating RMR by predictive equations was associated with errors, mainly overestimation [15,16], which could lead to an inadequate dietary prescription in patients with excess body fat.

It should be noted that there are few studies on this topic, especially in Latin America. The purpose of the study was to determine the most accurate predictive equation in the estimation of RMR in Chilean women with excess body fat, allowing a more accurate calculation of energy requirements, and facilitating the achievement of the objectives proposed by dietary prescription and nutritional therapy when indirect calorimetry equipment is not available. Therefore, the objective of the present study was to compare RMR measured by IC compared with RMR estimated by four predictive equations: FAO/WHO/UNU (1985), FAO/WHO/UNU (2004), Harris–Benedict, and Mifflin–St Jeor in young Chilean women with excess body fat.

## 2. Materials and Methods

### 2.1. Type of Study

This was an analytical cross-sectional study.

### 2.2. Population and Sample

The sample consisted of 41 young adult Chilean women 18 to 28 years of age with overnutrition. The participants met the following inclusion criteria: without hypermetabolic pathology, no anemia, stable body weight (±10%) in the last 3 months, without medication consumption that modified RMR prior to the measurement, no pathological history, regular menstrual cycles, and not pregnant or breastfeeding.

The present study was reviewed and approved by the Ethics and Biosecurity Committee of the Universidad del Bío-Bío and all the participants provided informed consent. The procedures were according to the ethical norms of the Declaration of Helsinki and the Council for International Organizations of Medical Sciences (CIOMS).

### 2.3. Anthropometric Measurements and Body Composition

To control the effect of the menstrual cycle on body composition and resting metabolic rate (RMR), all measurements were taken by the same evaluator between days 6 and 13 of the follicular phase of the menstrual cycle; this period was obtained from the analysis of each participant’s menstrual cycle [17]. The anthropometric measurements of weight and height [18] used the cutoff points established by the World Health Organization (WHO) [19].

Body composition was measured by using bioelectrical impedance analysis [20] with Bodystat 4000 equipment (Bodystat Quadscan, Southam, UK). The following conditions were verified prior to the measurement: not eating or drinking for 4 to 5 h; not exercising for 12 h; not menstruating; not consuming alcohol, nicotine/smoking, or caffeine for 24 h; wearing comfortable and light clothing; not wearing metallic accessories (jewelry, zippers, buttons); having urinated before the measurement; not having a pacemaker or metallic objects or plates acquired during surgery; not consuming certain medications (diuretics, corticosteroids); and not having renal failure. Each participant was lying supine with her arms and legs extended on the stretcher, and her right foot and hand were uncovered. Self-adhesive disposable electrodes were placed on the right hand and foot. The red conductor was placed on the right hand behind the knuckle of the middle finger and the black conductor on the wrist near the ulnar head. The red conductor was placed on the right foot behind the knuckle of the index toe and the black conductor on the ankle between the malleoli. The conductors were connected to the bioimpedance meter, which was switched on, and the requested data such as sex, age, weight, height, and hip circumference were recorded. Each participant was measured while keeping perfectly still and ensuring that no part of her body was touching any other part.

The nutritional status was diagnosed according to the percentage of body fat; body fat cutoff points were considered normal (20% to 30%), overweight (31% to 33%), and obese (>33%) [21].

### 2.4. Indirect Calorimetry (IC)

The indirect calorimetry method was applied (RMR IC) with the VMAX 29 N equipment (SensorMedics Corp., Yorba Linda, CA, USA), which belongs to the Energy Metabolism Unit of the Universidad del Bío-Bío. To estimate RMR from VO_2_ and VCO_2_ measurements, indirect calorimetry uses the Weir equation which relates RMR to oxygen consumption and carbon dioxide production. The equipment has a CO_2_ analyzer (accuracy: 0.02%; resolution: 0.01%) and also a sensor for O_2_ detection (accuracy: 0.02%; resolution: 0.01%).

The environment was controlled to ensure thermo-neutrality (20–24 °C) (Termio 31, Gesa, Urduliz, Spain) and a CO_2_ concentration < 3%. Prior to RMR measurement, the flow sensor was calibrated with a 3 L syringe, while the gas analyzers were calibrated with standardized gases (16% O_2_/4% CO_2_ and 26% O_2_) [22]. Each participant was checked for the absence of thyroid disease, was in the follicular phase of the menstrual cycle, had no anemia, and complied with the required fasting period (10–12 h) [23,24]. The following vital signs were also controlled: axillary body temperature < 37.0 °C and normal respiratory rate between 12 and 18 breaths/min.

The women had to rest for 30 min before the RMR IC measurement, which was taken first thing in the morning with the participant in the supine position, awake, and calm. Measurements were taken during approximately 30 min until reaching a steady state. The RMR IC was calculated by the equipment based on the measurement of O_2_ consumption and CO_2_ elimination. The steady state was defined as the first 5 min period with a coefficient of variation ≤ 10% for both the volumes of O_2_ and CO_2_ [25].

Test validity was ratified by the respiratory quotient value, which should show a normal physiological range from 0.7 to 1.0 [26]. It should also verify the fluctuation in the exchange of the volumes of CO_2_ (mL/ min) and O_2_ (mL/min) [27].

### 2.5. Predictive Equations

The results of the RMR IC were compared with the estimated RMR using the predictive equations of the FAO/WHO/UNU (1985), FAO/WHO/UNU (2004), Harris–Benedict, and Mifflin–St Jeor based on the actual weight of the participants (see Table 1). When RMR was estimated by the Harris–Benedict equation in a patient with obesity, the actual weight was used together with weight adjusted to 0.25 and 0.5 according to the recommended values [28]. This is expressed in Equations (1) and (2) [25,26].

(Body weight − ideal weight) × 0.25(1)

(Body weight − ideal weight) × 0.5(2)

The existence of adequacy, overestimation, and underestimation of the predictive equation was defined after calculating the percentage difference between RMR and RMR IC as expressed in Equation (3).

[(RMR − RMR IC)/(RMR IC × 100)](3)

Adequacy was understood to mean that the percentage difference between the estimated RMR and the RMR IC was within ±10%; that is, the result was adequate between 90% and 110%, and showed underestimation when it was <90% and overestimation when it was >110% [28,29,30,31].

### 2.6. Statistical Analysis

The normal distribution was verified by the Shapiro–Wilk test, and the variables were described by the median and percentiles. The Mann–Whitney and Kruskal–Wallis tests were used for comparison. We analyzed the concordance between the RMR predictive and the RMR IC according to the Bland–Altman method, the intraclass correlation coefficient (ICC), and coefficient of variation (CV). The information was processed with the STATA 16.0 software at a significance level of α < 0.05.

## 3. Results

The sample consisted of 41 young women from 18 to 28 years of age, whose nutritional status was overweight or obesity. In women with overweight, the medians (P50) for the age, weight, height, fat mass (%), free-fat mass (%), BMI, waist circumference, waist/hip circumference ratio, and RMR IC values were 22 y, 66.0 kg, 1.59 m, 30.9%, 69.1%, and 1.183.0 calories/day, respectively (Table 2).

In women with obesity, the medians (P50) for the age, weight, height, fat mass (%), free-fat mass (%), BMI, waist circumference, waist/hip circumference ratio, and RMR IC values were 23.5 y, 67.5 kg, 1.58 m, 35.3%, 65.1%, and 1.192.0 calories/day, respectively. Women with overweight and obesity showed statistically significant differences in the fat mass (%) and free-fat mass (%) variables (*p* < 0.05) (Table 2).

In women with overweight, the RMR IC showed a median of 1.183 calories/day, and 25% (P25) of them expended < 1116 calories/day and 75% (P75) < 1295 calories/day. The RMR estimated by the FAO/WHO/UNU (1985) equation showed a median of 1466.2 calories/day, and 25% of subjects obtained values < 1392.7 calories/day and 75% < 1504.4 calories/day. When using the FAO/WHO/UNU (2004) equation, the RMR median was 1494.2 calories/day, and 25% of the subjects had an RMR < 1427.5 calories/day and 75% < 1760.9 calories/day. The Harris–Benedict equation showed a median of 1475.7 calories/day, and 25% of the women showed values < 1434.6 calories/day and 75% < 1500.7 calories/day. When applying the Mifflin–St Jeor equation, a median of 1386.0 calories/day was obtained, and 25% of the subjects showed values < 1340.7 calories/day and 75% < 1439.8 calories/day. There were statistically significant differences between the values obtained by the predictive equations and RMR IC (*p* < 0.001). In the group of women with overweight, the FAO/WHO/UNU (1985), FAO/WHO/UNU (2004), Harris–Benedict, and Mifflin–St Jeor formulae overestimated the RMR IC by 283.2, 311.2, 292.7, and 203.0 calories/day, respectively (a detailed analysis is available in Figure S1: Resting metabolic rate (RMR) measured by indirect calorimetry (IC) vs. estimation by predictive equations in overweight women).

In the women with obesity, the RMR IC showed a median of 1192 calories/day, and 25% of them expended < 1115 calories/day and 75% < 1346 calories/day. When evaluating RMR estimated by the FAO/WHO/UNU (1985) equation, the median was 1488.2 calories/day, and 25% of the subjects showed RMR < 1392.7 calories/day and 75% < 1605.8 calories/day. The FAO/WHO/UNU (2004) equation results showed a median of 1605.3 calories/day, and 25% of the women obtained values < 1477.9 calories/day and 75% < 1790.4 calories/day. The median using the Harris–Benedict equation was 1471.5 calories/day, and 25% of the subjects had an RMR < 1420.7 calories/day and 75% < 1563.9 calories/day. The Harris–Benedict adjusted weight (0.25) equation showed a median of 1368.1 calories/day, and 25% of the women obtained values < 1336.8 calories/day and 75% < 1420.8 calories/day. The Harris–Benedict adjusted weight (0.5) equation showed a median of 936.8 calories/day, and 25% of the subjects obtained an RMR < 920.0 calories/day and 75% < 1378.3 calories/day. The estimation by the Mifflin–St Jeor formula showed a median of 1368.1 calories/day, and 25% of the subjects had values < 1307.1 calories/day and 75% < 1480.0 calories/day. There was a statistically significant difference between the values estimated by the predictive equations and the RMR IC (*p* < 0.001). In the group of women with obesity, the FAO/WHO/UNU (1985), FAO/WHO/UNU (2004), Harris–Benedict, Harris–Benedict adjusted weight (0.25), and Mifflin–St Jeor predictive equations overestimated the RMR IC by 296.7, 413.8, 280.0, 176.6, and 176.6 calories/day, respectively. Additionally, the Harris–Benedict adjusted weight (0.5) equation underestimated the RMR IC by 254.7 calories/day (Figure S2: Resting metabolic rate (RMR) measured by indirect calorimetry (IC) vs. estimation by predictive equations in women with obesity, Supplementary Materials).

**Figure 1:**
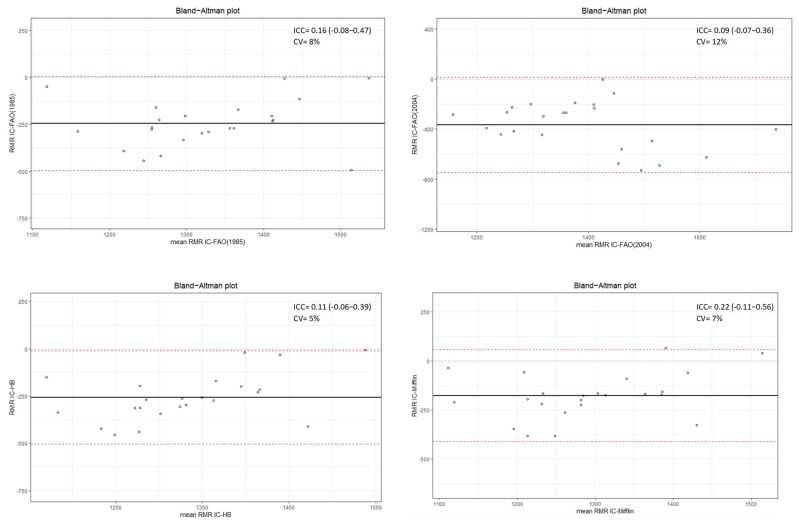
Bland–Altman plot for comparison of predicted and reference resting metabolic rate in overweight women. RMR IC: indirect calorimetry; FAO 2004: FAO/WHO/UNU (2004); FAO 1985: FAO/WHO/UNU (1985); HB: Harris–Benedict; Mifflin: Mifflin–St Jeor; ICC: intraclass correlation coefficient; CV: coefficient of variation.

**Figure 2:**
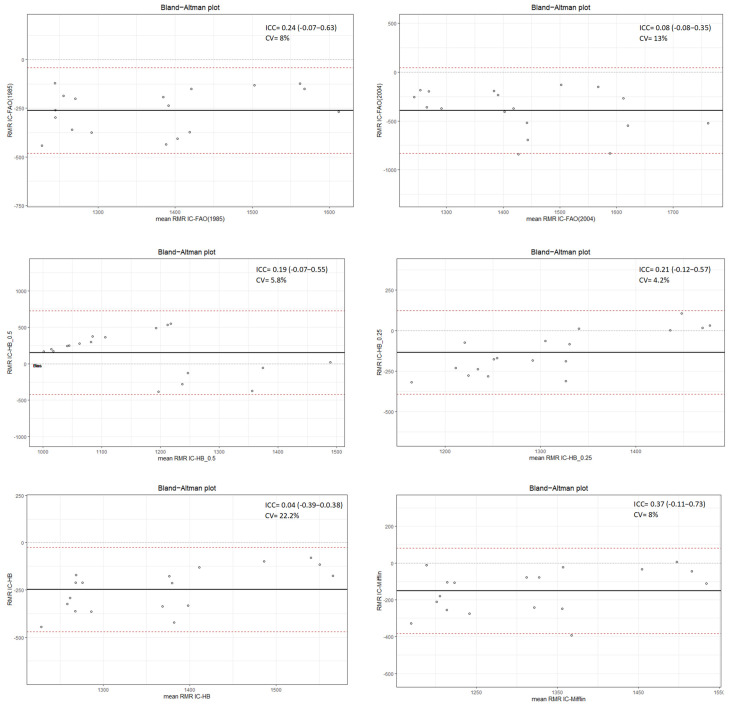
Bland–Altman plot for comparison of predicted and reference resting metabolic rate in women with obesity. RMR IC: indirect calorimetry; FAO 2004: FAO/WHO/UNU (2004); FAO 1985: FAO/WHO/UNU (1985); HB: Harris–Benedict; HB_0.25: Harris–Benedict adjusted weight at 0.25; HB_0.5: Harris–Benedict adjusted weight at 0.5; Mifflin: Mifflin–St Jeor; ICC: intraclass correlation coefficient; CV: coefficient of variation.

The Figure 1 and Figure 2 show the agreement of the predictive methods with respect to the reference (CI). It is possible to observe that in none of the methods there is concordance in the RMR estimations. In relation to overweight women, the lowest concordance was for the FAO/WHO/UNU (2004) equation (CV = 12%), while for women with obesity it was Harris–Benedict adjusted weight at 0.5 (CV = 22%). In both groups the Mifflin–St Jeor equation exhibits the lowest range of variability for the estimation of RMR considering a cut-off point of 200 Kcal.

In the group of women with overweight, the Mifflin–St Jeor predictive equation provided the best adequacy (90% to 110%) in 30.4% of the women; however, it overestimated the RMR IC in 69.5%. The Harris–Benedict equation showed a relevant overestimation in 86.9% of the participants (Table 3).

In the group of women with obesity, the Mifflin–St Jeor predictive equation provided the best adequacy (90% to 110%) in 55.5% of the women; however, it overestimated the RMR IC in 44.4%. The Harris–Benedict, FAO/WHO/UNU (1985), and FAO/WHO/UNU (2004) equations showed higher overestimation by 77.7%, 83.3%, and 88.8%, respectively. Finally, the Harris–Benedict adjusted weight (0.5) equation underestimated the RMR IC in 66.7% of the women (Table 4).

## 4. Discussion

It is common in the nutrition consultation to estimate the RMR by predictive equations and then determine the total energy requirement using the factorial method [11]. However, applying predictive equations to estimate RMR to groups of subjects with different characteristics such as ethnicity, body weight, body composition, height, and age can increase the estimation error. Most published articles that analyze the behavior of these equations in overnutrition use the BMI (body mass index) to classify the nutritional status; BMI is a low sensitivity indicator to evaluate body fat [32]. Therefore, the nutritional status in the present study was classified by body composition analysis to avoid inaccuracies when analyzing the estimation of RMR by predictive equations in women with overweight or obesity.

Studies have shown that predictive equations can overestimate or underestimate the RMR [33,34], leading to a caloric excess or deficit, respectively, and inadequate nutritional recommendations. It has also been reported that their accuracy or predictive ability decreases in subjects with excess body fat [35,36]. The study addressed the equations most frequently used by researchers and in routine clinical practice. The FAO/WHO/UNU equation is recommended by the World Health Organization and was validated at the FAO/WHO/UNU Expert Consultative Meeting in 2001, the Harris–Benedict equation is the pioneer equation referenced in books and scientific articles, and the Mifflin–St Jeor equation is recommended by the American Dietetic Association and the Dietitians of Canada.

The RMR IC median was similar between women with overweight and obesity (p = 0.429) with 1183 calories/day and 1192 calories/day, respectively. In women with overweight, the FAO/WHO/UNU (1985), FAO/WHO/UNU (2004), Harris–Benedict, and Mifflin–St Jeor predictive equations overestimated (>110%) the RMR IC (*p* = 0.001). The FAO/WHO/UNU 2004 equation showed the highest overestimation of 311.2 calories, while the Mifflin–St Jeor equation showed the lowest overestimation of 203.0 calories; these values are similar to those reported in other studies [29,37]. For the women with obesity, the predictive equations also overestimated (>110%) RMR IC. The FAO/WHO/UNU (2004) equation showed the highest overestimation of 413.8 calories, while the Mifflin–St Jeor equation showed the lowest overestimation of 176.6 calories.

The overestimation of RMR IC obtained by the FAO/WHO/UNU (1985) equation has been reported in other studies [34,38,39]. This could be explained in the present study by the ethnic and body composition differences of the participating women. It should be considered that this equation resulted from a study conducted by Scholfield that involved North American and European subjects, which had no data for subjects from developing countries, limited ethnic and geographic variation, and 45% of the subjects were Italians with a higher RMR/kg of body weight than the rest of the participants [11]. As for the overestimation resulting from the Harris–Benedict equation, it can also be explained by the ethnicity and nutritional status of the population from which it was derived. It was based on a study of 239 white subjects of whom 103 were women; the ethnic origin of the participants was unspecified, and all of them had normal body weight [10].

The FAO/WHO/UNU (1985) and Harris–Benedict predictive equations overestimated RMR IC in subjects with overnutrition; this concurs with findings reported by Carrasco et al. in a study conducted with Chilean women with obesity [40]. It also coincides with the description by Frankenfield et al., who reported that the Mifflin–St Jeor equation showed the lowest overestimation [28], which is similar to our findings. In addition, it has been reported that the Mifflin–St Jeor equation has a low accuracy (46% to 60%) for estimating RMR in women with obesity, and there is a clear trend for underestimating RMR in Mexican [41] and Brazilian women [42]. Significant errors and limitations have been reported when this equation is generalized to certain age and ethnic groups [28].

The Mifflin–St Jeor equation showed the lowest overestimation of RMR in both women with overweight and obesity, so it is recommended as the most reliable, especially in subjects with obesity. This is consistent with what has been reported in other studies [37,43,44]. The equation was derived from measurements of RMR by indirect calorimetry that involved 251 men and 247 women, with 47% of the participants exhibiting overnutrition and BMI between 30 and 42 kg/m^2^; unfortunately, the ethnic composition of the subjects was not reported [12]. The Mifflin–St Jeor equation showed the best adequacy for RMR IC (90% to 110%), which is similar to that reported in other studies [28,45]. However, it is not without some inaccuracies because it overestimated RMR IC in 69.5% and 44.4% of women with overweight and obesity, respectively. These values are much higher than those reported in other studies, in which the percentage of subjects with RMR IC overestimated reached 20% [28,37,44]. As for the FAO/WHO/UNU (1985) and FAO/WHO/UNU (2004) equations, the first equation showed a relatively better adequacy in our study. This is in line with the decision made by experts at the meeting held in Rome in 2001 at which the use of the FAO/OMS/UNU (1985) equation was maintained [45].

In patients with obesity, it is not clear which weight to use to estimate RMR by predictive equations, whether actual, ideal, or adjusted weight, despite the recommendation to use actual weight [46]. To reduce the risk of overestimation, specifically with the Harris–Benedict equation, it has been recommended not to use the real weight of individuals with obesity, but rather the adjusted weight [28] at 25% or 50% [29,30]. This is because the increase in body weight not only increases body fat mass but also fat-free mass, which is metabolically more active and accounts for approximately 25% of the increase in total body weight [47]. The estimation of RMR by the Harris–Benedict equation using adjusted weight at 25% overestimated RMR IC by 176.6 calories on average, as opposed to the underestimation reported in another study [29]. Conversely, the estimation with adjusted weight at 50% showed a significant underestimation of 254.7 calories on average in 66.7% of women with obesity.

Any excess energy intake is stored in the body, and a positive energy balance between 6600 and 8000 calories produces a 1 kg gain in body weight [48]. Given the influence of genetic factors on body composition [49] and the nutritional status of the population in which predictive equations are applied, equations that do not respond to ethnic characteristics can lead to an inadequate energy recommendation and contribute to an undesirable change in body weight over the medium to long term.

One of the strengths of the present study was that it carefully controlled the effect of several potentially important misleading factors when measuring RMR IC. Examples include taking the measurement during the follicular phase of the women’s menstrual cycle, absence of relevant medical conditions, and avoiding possible thermogenetic effects of food, caffeine, and nicotine.

Given the high prevalence of overweight and obesity in Chilean women, there is concern that the estimation of RMR by predictive equations in clinical settings is inaccurate and exceeds the real energy needs of the individuals to whom the estimation is applied. If it is not feasible to measure the RMR by IC, the Mifflin–St Jeor equation could be an option for the group of overweight women or those with obesity because it overestimated less and has better adequacy. However, it is also very important to clarify that this method is not free of inaccuracies [28]. Finally, further studies are required to develop and validate predictive equations that respond to the ethnic characteristics of the Chilean population. These can be applied to treat and control body weight in women with excess body fat without the risk of overestimating or underestimating RMR and thus their energy needs.

## 5. Conclusions

In conclusion, the analyzed predictive equations overestimated the resting metabolic rate by indirect calorimetry (RMR IC) in a group of young Chilean women with overweight or obesity (30.6% to 36% fat mass). However, the estimation by means of the Mifflin–St Jeor equation using real weight could be the recommended option among the equations analyzed both for sport nutrition or nutritional therapy, as it presents the lowest overestimation and the best adequacy, even though it is not exempt from inaccuracy. The Harris–Benedict equation with adjusted weight (0.50) should not be recommended, which showed a relevant and worrisome underestimation.

## Figures and Tables

**Table 1:** Predictive equations to estimate the resting metabolic rate.

Equation	Subjects	Nutritional Status	Age	Equation for Female Subjects
FAO (1985)	247	Normal Overweight Obesity	19–82	(14.7 × W) + 496 (18 to 30 years)
FAO (2004)	247	Normal Overweight Obesity	19–82	(14.818 × W) + 886.6 (18 to 30 years)
Harris–Benedict	239	Normal	15–74	(9.563 × W) + (1.84 × H) − (4.676 × E) + 655.09
Mifflin–St Jeor	498	Normal Overweight Obesity	19–78	(9.99 × W) + (6.25 × H) − (5 × A) − 161

W: body weight (kg); H: height in cm; A: age (years).

**Table 2:** Characterization of women with excess body fat.

Nutritional Status	Percentile (P)	Age (Years)	Weight (kg)	Height (m)	Fat Mass (%)	Fat-Free Mass (%)	BMI (kg/m^2^)	Waist Circumference	Waist/Hip Ratio	RMR IC (Calories)
Overweight (*n* = 23)	P25	21	61.0	1.57	30.6	67.7	24.0	74.5	0.76	1116
	P50	22	66.0	1.59	30.9	69.1	25.1	76.5	0.78	1183
	P75	23	68.6	1.64	32.0	69.4	26.4	81.0	0.81	1295
Obesity (*n* = 18)	P25	22	61.0	1.55	34.3	64.6	25.4	82.0	0.75	1115
	P50	23.5	67.5	1.58	35.3	65.1	26.9	83.6	0.78	1192
	P75	26	75.5	1.60	36.0	66.3	29.5	88.0	0.84	1346
*p*-value		0.031	0.226	0.252	<0.001	<0.001	0.0191	<0.001	0.4732	0.5993

Mann–Whitney test; RMR IC: resting metabolic rate by indirect calorimetry.

**Table 3:** Adequacy, overestimation, or underestimation of the resting metabolic rate (RMR) estimated by predictive equations for RMR by indirect calorimetry (RMR IC) in women with overweight.

Predictive Equation	Adequacy (90% to 110%)	Overestimation (>110%)	Underestimation (<90%)
Mifflin–St Jeor	30.4	69.5	0
Harris–Benedict	13.0	86.9	0
FAO (1985)	17.3	82.6	0
FAO (2004)	8.6	91.3	0

**Table 4:** Adequacy, overestimation, or underestimation of the resting metabolic rate (RMR) estimated by predictive equations for RMR by indirect calorimetry (RMR IC) in women with obesity.

Predictive Equation	Adequacy (90% to 110%)	Overestimation (>110%)	Underestimation (<90%)
Mifflin–St Jeor	55.5	44.4	0
Harris–Benedict	22.2	77.7	0
Harris–Benedict AW 0.25	44.4	55.5	0
Harris–Benedict AW 0.50	11.1	22.2	66.7
FAO (1985)	16.6	83.3	0
FAO (2004)	11.1	88.8	0

AW 0.25: adjusted weight 0.25; AW 0.50: adjusted weight 0.50.

## Data Availability

The data presented in this study are available on request from the corresponding author. Data is not publicly available due to privacy.

## Supplementary Materials

The following supporting information can be downloaded at https://www.mdpi.com/article/10.3390/metabo13020188/s1, Figure S1: Resting metabolic rate (RMR) measured by indirect calorimetry (IC) vs. estimation by predictive equations in overweight women, Figure S2: Resting metabolic rate (RMR) measured by indirect calorimetry (IC) vs. estimation by predictive equations in women with obesity.
