# Supplementary material for: Predictive Equations Overestimate Resting Metabolic Rate in Young Chilean Women with Excess Body Fat
Source: Metabolites. 2023 Jan 27;13(2):188. doi: 10.3390/metabo13020188 (PMC9964988; doi:10.3390/metabo13020188)
Supplement: Supplementary file 1 [file metabolites-13-00188-s001.zip › metabolites-2147465-supplementary.pdf]

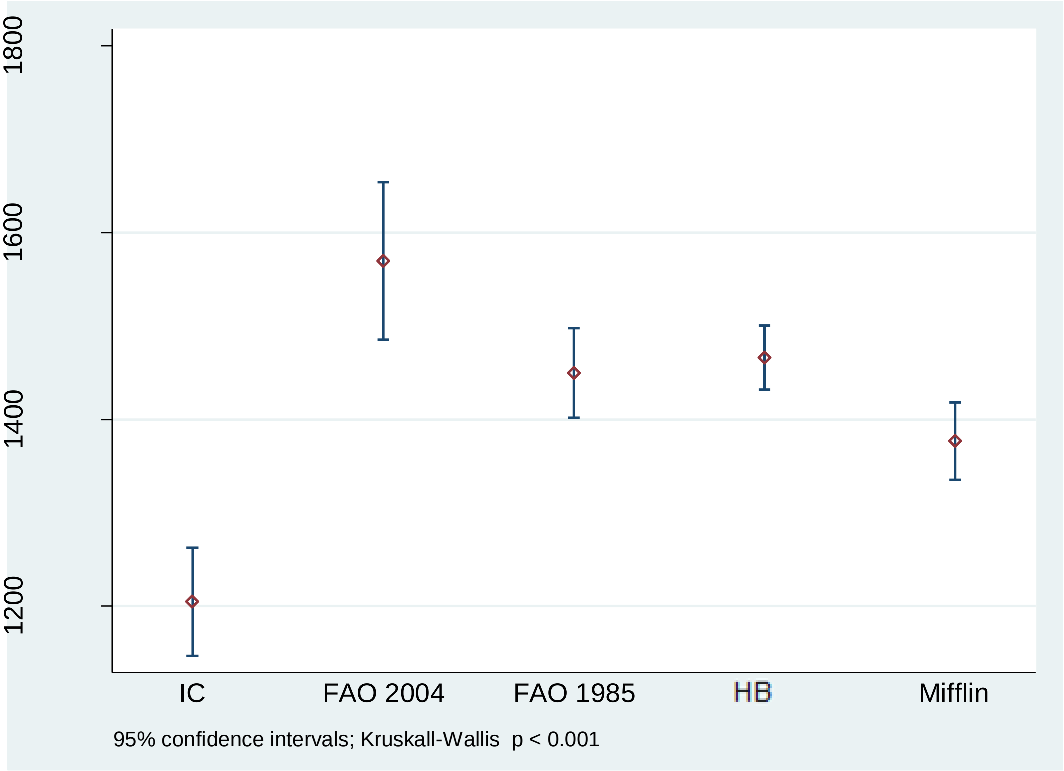

Figure S1. Resting metabolic rate (RMR) measured by indirect calorimetry (IC) vs. estimation by predictive equations in overweight women. IC: RMR IC; FAO 2004: FAO/WHO/UNU (2004); FAO 1985: FAO/WHO/UNU (1985); HB: Harris-Benedict; Mifflin: Mifflin-St Jeor.

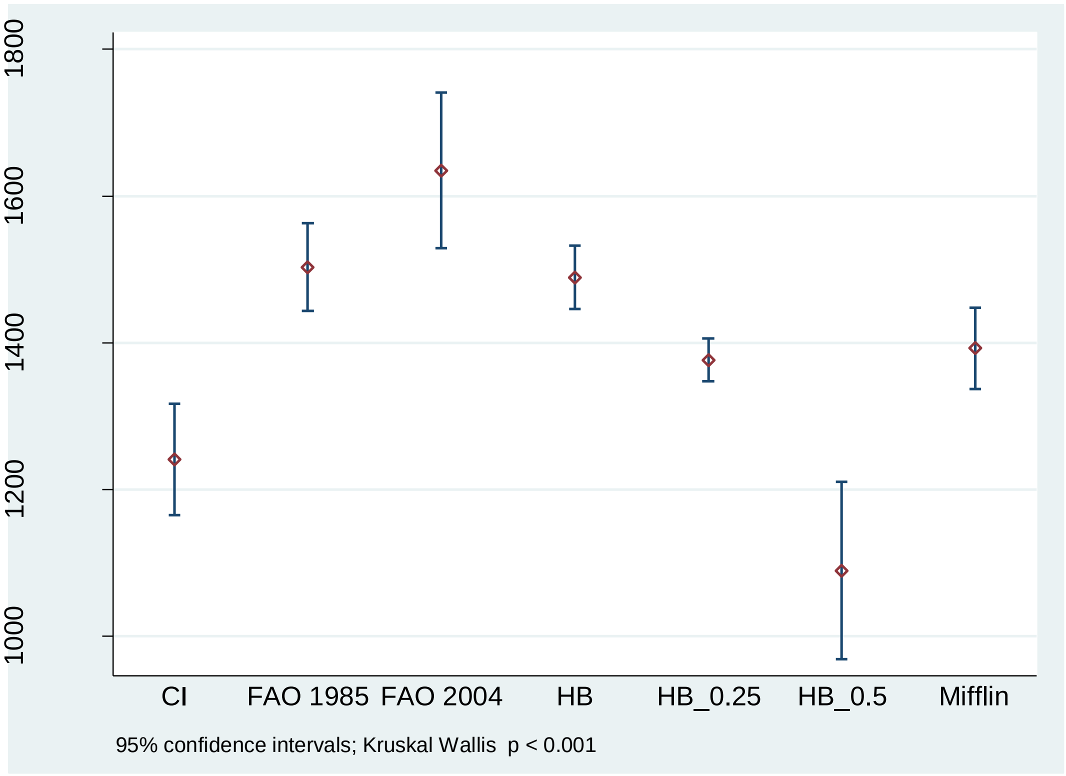

Figure S2. Resting metabolic rate (RMR) measured by indirect calorimetry (IC) vs. estimation by predictive equations in women with obesity. IC: RMR IC; FAO 2004: FAO/WHO/UNU (2004); FAO 1985: FAO/WHO/UNU (1985); HB: Harris-Benedict; H_0.25: Harris-Benedict adjusted weight at 0.25; H_0.5: Harris-Benedict adjusted weight at 0.5; Mifflin: Mifflin-St Jeor.
